# Graphene Oxides (GOs) with Different Lateral Dimensions and Thicknesses Affect the Molecular Response in *Chironomus riparius*

**DOI:** 10.3390/nano13060967

**Published:** 2023-03-07

**Authors:** Raquel Martin-Folgar, Adrián Esteban-Arranz, Viviana Negri, Mónica Morales

**Affiliations:** 1 Grupo de Biología y Toxicología Ambiental, Departamento de Física Matemática y de Fluidos, Facultad de Ciencias, UNED, Urbanización Monte Rozas, Avda. Esparta s/n, Crta. de Las Rozas al Escorial Km 5, 28232 Madrid, Spain; 2 Instituto de Ciencia y Tecnología de Polímeros (ICTP-CSIC), C/Juan de la Cierva 3, 28006 Madrid, Spain; 3 Departamento de Ciencias de la Salud de la Universidad Europea de Madrid (UEM), C/Tajo, Villaviciosa de Odón, 28670 Madrid, Spain

**Keywords:** graphene oxide, freshwater ecotoxicology, oxidative stress, *Chironomus riparius*, molecular response

## Abstract

Graphene oxide (GO) materials possess physicochemical properties that facilitate their application in the industrial and medical sectors. The use of graphene may pose a threat to biota, especially aquatic life. In addition, the properties of nanomaterials can differentially affect cell and molecular responses. Therefore, it is essential to study and define the possible genotoxicity of GO materials to aquatic organisms and their ecosystems. In this study, we investigated the changes in the expression of 11 genes in the aquatic organism *Chironomus riparius* after 96 h of exposure to small GOs (sGO), large GOs (lGO) and monolayer GOs (mlGO) at 50, 500 and 3000 μg/L. Results showed that the different genes encoding heat shock proteins (hsp90, hsp70 and hsp27) were overexpressed after exposure to these nanomaterials. In addition, ATM and NLK—the genes involved in DNA repair mechanisms—were altered at the transcriptional level. DECAY, an apoptotic caspase, was only activated by larger size GO materials, mlGO and lGO. Finally, the gene encoding manganese superoxide dismutase (MnSOD) showed higher expression in the mlG O-treated larvae. The lGO and mlGO treatments indicated high mRNA levels of a developmental gene (FKBP39) and an endocrine pathway-related gene (DRONC). These two genes were only activated by the larger GO materials. The results indicate that larger and thicker GO nanomaterials alter the transcription of genes involved in cellular stress, oxidative stress, DNA damage, apoptosis, endocrine and development in *C. riparius*. This shows that various cellular processes are modified and affected, providing some of the first evidence for the action mechanisms of GOs in invertebrates. In short, the alterations produced by graphene materials should be further studied to evaluate their effect on the biota to show a more realistic scenario of what is happening at the molecular level.

## 1. Introduction

Graphene oxide (GO) and its derivatives are carbon nanomaterials with huge potential in various fields, such as biomedicine, textiles, drug delivery, catalysis [1] and adsorption [2,3]. However, their manufacture and application involve their release into the environment [4]. This could cause a great risk to the health of organisms as well as ecosystems. Several in vivo studies have reported the persistence and distribution of GO in living organisms. The cytotoxicity induced by graphene materials was tested in various species, such as prokaryotes, bacteria, viruses, plants, invertebrates and mammals, including human and animal cells [5,6,7,8,9,10]. Several studies suggest that the size of the material will determine how it interacts with cells [11,12,13]. A previous work carried out with diverse cell lines has shown that GO with different side-chain dimensions has been able to cross the cell membrane [2]. They were even detected in lysosomes [14]. The proven potential of these nanomaterials to cross cell membranes makes it necessary to analyze their effects at the molecular level and to study the possible altered cellular metabolic pathways due to their toxicity.

*Chironomus riparius* is a freshwater dipteran used as a bioindicator in ecotoxicology tests [15,16,17]. Recent results from our group demonstrated that the exposure to and subsequent ingestion of GOs by chironomids causes accumulation in the intestinal tract, activating the superoxide dismutase (SOD) and lipid peroxidation (LPO) [18]. Martinez de Paz et al., 2019 [19], demonstrated that the *C. riparius* larvae, when treated with multi-walled carbon nanotubes (MWCNT), showed accumulation in the intestinal tract. This was shown to affect its gene expression in DNA repair mechanisms, cell stress response and apoptosis [19]. However, studies on this subject are very scarce. Based on the information available at present, we could only assume that exposure to graphene oxide nanomaterials can affect gene expression in *C. riparius*. The interactions of insects and graphene-based materials (GBMs) in aquatic ecosystems have been little studied and information on their effects is scarce [18,19,20]. Studies with other aquatic invertebrate species, such as *Daphnia magna* and *Caenorhabditis elegans*, suggest that GO could induce acute toxicity, bioaccumulation and oxidative stress [21,22,23].

Currently, there are virtually no studies that are focused on the changes in gene expression in aquatic species, including invertebrates [22,23] and fish [21,24]. Moreover, we hypothesize that GOs with different side-chain dimensions and thickness [4] could produce changes in the gene expression in *C. riparius*. These modifications may be detected 96 h after exposure. They can point to the influence of some physicochemical properties of nanomaterials on aquatic organisms. To address this work, 11 genes related to several fundamental invertebrate metabolic pathways were studied, such as heat stress response (hsp90, hsp70, hsp60 and hsp27), DNA repair (X-ray repair cross complementing 1 [XRCC1], ataxia-telangiectasia mutated [ATM], NEMO-like kinase [NLK] and death executioner caspase related to Apopain/Yama [DECAY]), oxidative stress response (manganese superoxide dismutase [SOD Mn]), endocrine system (death regulator Neddd2-like caspase [DRONC]) and development (FKBP39). These genes could be biomarkers of damage. *C. riparius* larvae will be treated with concentrations of 50, 500 and 3000 µg/L for 96 h.

## 2. Materials and Methods

### 2.1. Synthesis and Characterisation of GO Materials

Three GO samples with different size and lateral dimensions were synthesized to evaluate their effect in *C. riparius*: large graphene oxide (lGO), monolayer large graphene oxide (mlGO) and small graphene oxide (sGO). lGO was obtained according to the protocol developed by Jasim et al. (2016). [25] Graphite powder was treated with sulphuric acid and sodium nitrate in contact with an ice bath, followed by drop-by-drop addition of the oxidising agent, potassium permangate, until a homogenous green solution was achieved. Water was then slowly added, and the mixture was stirred at 98 °C for 30 min. The reaction was stopped with the addition of hydrogen peroxide. The day after the synthesis, the graphitic carbon was removed and lGO was purified by centrifugation. mlGO was recollected from lGO washing fraction and separated following methodology outlined elsewhere [3]; sGO was obtained by an experimental approach published elsewhere [26], which was based on the controlled ultrasound of lGO for 5 min and then purified by centrifugation.

**Figure 1:**
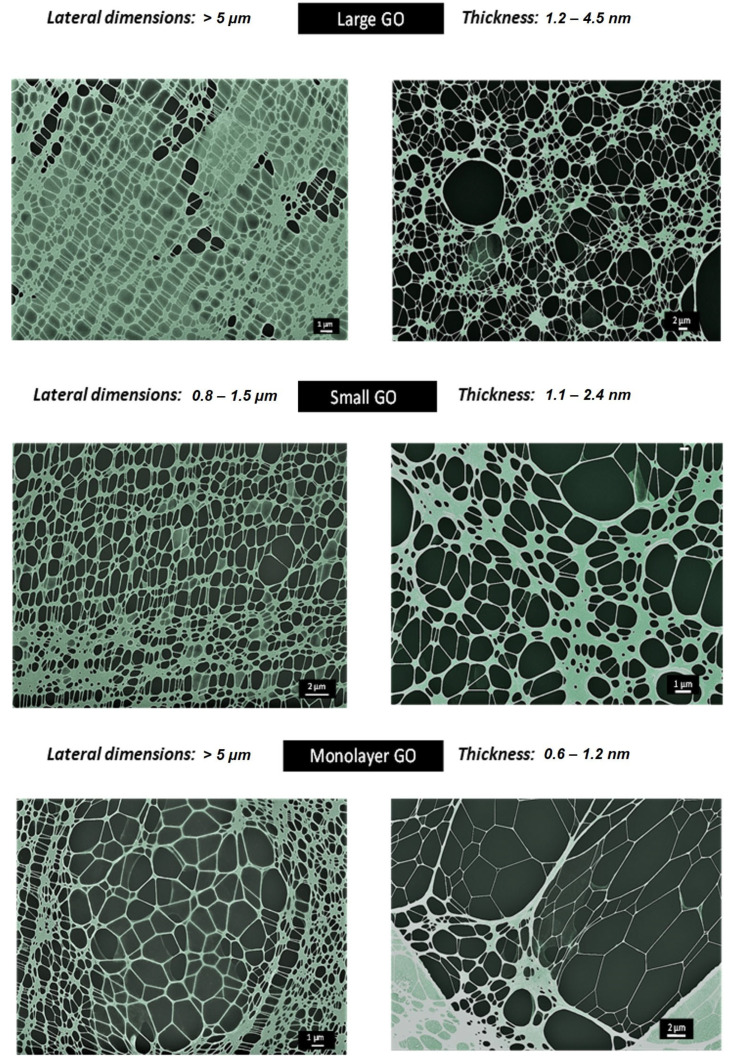
STEM micrographs of the three GO materials. It can be seen that they present flat structures typical of this type of nanomaterial. In addition, lateral dimension and thickness values have been incorporated.

Surface functional groups of GO materials were analyzed by Fourier transform infrared spectroscopy (FTIR) employing a Spectrum Two Fourier transform infrared spectrometer (PerkinElmer, Waltham, MA, USA) with a zinc selenide (ZnSe) crystal (Supplementary Figure S1). Wave number spectra range was set from 4000 to 450 cm^−1^, with a 4 cm^−1^ resolution and 150 scans per sample. Turbiscan TM Lab Expert stability analyzer (Toulouse, France) was used to evaluate colloidal stability in the culture media of *C. riparius* for 24 h (Supplementary Figure S2). Results were presented as Turbiscan Stability Index (TSI) values. Scanning Transmission Electronic Microscopy (GeminiSEM 500 in STEM mode, ZEISS, Oberkochen, Germany) was also used for the determination of their lateral dimensions (Figure S3 and Figure 1). Raman experiments were conducted at 50× by a Renishaw InVia spectrometer (Wotton-under-Edge, UK) with a 633 nm 0.1% power laser. Spectra were recorded from 1000 to 3250 cm^−1^. Spectra were normalized by the intensity of graphitic (G) band in the OriginPro 8.5 software (OriginLab, Northampton, MA, USA) (Supplementary Figure S4). X-ray diffraction experiments were carried out in a Philips X’Pert MPD diffractometer (Malvern Panalytical, Malvern, UK) with Cu Kα1 (1.54056 Å) radiation at 40 mA and 40 kV. Graphene oxide dispersions (3000 µg/mL) were placed in a cylindrical vial (30 mL) prior their measurement (Supplementary Figure S5).

### 2.2. Animals and Treatments

The model organism used was the fourth instar *C. riparius* larvae. The organisms were cultured following the guidelines indicated for conducting toxicity tests (OECD, 2010, 2011). Larvae from the midge *C. riparius* were grown in culture medium (0.5 mM CaCl_2_, 1 mM NaCl, 1 mM MgSO_4_, 0.1 mM NaHCO_3_, 0.025 mM KH_2_PO_4_) supplemented with nettle leaves, commercial fish food and cellulose tissue. Cultures were maintained under constant aeration at 20 °C and under standard 16-h light–8-h dark cycle. Larvae were exposed in glass recipients (200 mL) and covered with aluminium foil to avoid photodecomposition. Small GO (sGO), large GO (lGO) and monolayer GO (mlGO) nanoparticles were diluted in 50 mL of culture medium to obtain the concentrations selected for exposures (50, 500 and 3000 µg/L). They were then incubated for 96 h. The medium was renewed every 48 h, and the larvae were fed at 48 h [27]. The exposure concentrations of GOs in this study were based on previous results in aquatic organisms [14,28,29]. Ambient concentrations in the aquatic environment have been reported in the range of 1–1000 µg/L. We selected two concentrations within this range and one above it (50, 500 and 3000 µg/L). Each treatment consisted of three replicates, and three independent experiments were performed in each analysis using samples from three different egg masses. Larvae were stored at −80 °C until RNA isolation was carried out. Concentrations of 50, 500 and 3000 µg/L for small GO (sGO), large GO (lGO) and monolayer GO (mlGO), respectively, were used to study the xenobiotic potential of these nanomaterials in the chosen species. The exposures were maintained for 96 h, similar to the conditions employed in the previous study [30]. Three separate experiments were conducted, and each treatment was carried out in triplicate with pools of five larvae each. An experimental control of unexposed larvae was used in parallel to the treated individuals.

### 2.3. RNA Extraction and Complementary DNA Synthesis

The pool of five larvae were homogenized with 300 µL of Trizol (Sigma-Aldrich, Burlington, MA, USA) and centrifuged for 15 min at 10,000× *g* and 4 °C. RNA was recovered in the aqueous phase and 60 µL of chloroform was added. Then, it was mixed and incubated for 3 min at room temperature. The sample was centrifuged 15 min at 10,000× *g* and 4 °C. The upper phase was recovered and precipitated with 150 µL of isopropyl alcohol at RT for 10 min and centrifuged for 10 min at 10,000× *g* and 4 °C. The supernatant was discarded, and the pellet was washed with 1 mL of 75% ethanol. Finally, the sample was centrifugated for 5 min at 10,000× *g* and 4 °C. The supernatant was discarded and the ethanol was removed with a pipette, and the RNA was resuspended in 44 µL of DEPC water [27]. RNAase-free DNAase I (Roche, Basel, Switzerland) was used to treat the extracted total RNA. 44 µLof RNA was incubated with 5 µL of 10X DNase buffer, and 1 µL of RNAse-free DNAase I for 60 min at 37 °C. Afterwards, the enzyme was removed with phenol-chloroform-isoamyl alcohol extraction using Gel Phase Lock Light tubes (5PRIME QuantaBio, Beverly, MA, USA). The aqueous phase was precipitated with 1 volume of isopropyl alcohol and washed with 75% ethanol (1 mL). Finally, the RNA was resuspended in 50 µL DEPC water and stored at −80 °C. The RNA was quantified with spectrophotometry and checked by agarose gel electrophoresis (1.5%).

To synthesize complementary DNA (cDNA), 500 ng of total RNA, 500 ng of oligonucleotide (polyT) (Invitrogen, Waltham, MA, USA), 1 µL of dNTPs (10 mM) (Biotools, Madrid, Spain) and DEPC water were used to create a final volume of 20 µL. The mix was incubated for 5 min at 65 °C and cooled on ice. Then, the samples were centrifuged and 4 µL of First-strand Buffer 5X (Invitrogen, Waltham, MA, USA), 2 µL DTT (Invitrogen, Waltham, MA, USA), and 0.5 µL of M-MLV enzyme (Invitrogen, Waltham, MA, USA) were added. The samples were incubated 50 min at 37 °C. The reaction was inactivated for 15 min at 70 °C. The cDNA was stored at −20 °C.

### 2.4. Real-Time Polymerase Chain Reaction

The previously extracted cDNA was used as a template in real-time polymerase chain reaction (PCR) to analyze the mRNA expression. Real-time polymerase chain reaction (PCR) was performed using SsoFast EvaGreen Supermix (BioRad, Hercules, CA, USA) in a CFX96 thermocycler (BioRad, Hercules, CA, USA). The conditions employed in the reaction were performed using 50 ng cDNA, oligonucleotides forward and reverse (2.5 µM) and 2X solution of dNTPs and reaction buffer (Morales et al., 2020). [31] The sequences of the oligonucleotides used are shown in Table 1. The reference genes in this study were glyceraldehyde 3-phosphate dehydrogenase (*GAPDH*) and ribosomal protein L13 (*rpL13*). The samples were analyzed in duplicate where two replicates of each plate were run. Amplification conditions consisted of 30 s at 95 °C followed by 39 cycles of 95 °C for 5 s, 58 °C for 15 s and 65 °C for 10 s including the plate readout and a denaturation curve consisting of 0.5 °C increments from 65 to 95 °C for 5 s, each with the plate readout.

### 2.5. Statistical Analysis

The statistical study of the expression data obtained by real-time PCR was performed. Untreated and GO-exposed larvae were compared using analysis of variance (ANOVA) with Dunnett’s multiple comparison tests. Normality and homogeneity of variances were calculated by Kolmogorov-Smirnov and Levene tests, respectively. All statistical tests were performed using SPSS 22.0 (IBM, North Castle, NY, USA). The results were considered significant with *p* < 0.05. The results are expressed as the mean ± standard error of the mean (SEM) of three experiments [31]. In the case of characterization results of nanomaterials, standard deviation has been incorporated for Turbiscan analyses (Supplementary Figure S2) and Raman spectra (Supplementary Figure S4).

## 3. Results and Discussion

The literature regarding the effects of GO on aquatic species at the molecular level is very scarce, but indications are beginning to emerge and show that graphene nanomaterials may be considered as cytotoxic.

Previous studies in Zebrafish embryos have shown that GO materials in suspension at concentrations between 50 and 100 mg/L undergo agglomeration over time and this probably influences their toxicity [24]. In addition to this, they can penetrate the cell membrane and interact with the cellular structures. Although the mechanism of their toxicity is not known, LPO, increased reactive oxygen species (ROS), nutrient depletion and inflammation are some of the toxicity mechanisms most triggered by graphene-based nanomaterials among aquatic organisms [9].

This study evaluated the expression of 11 genes in the aquatic insect *C. riparius* that was exposed to GO materials with different lateral dimensions and thickness. These materials are currently used in different areas of the industry (medicine, electronics, energy, etc.). The materials were previously synthesized and characterized [18]. Their characterisation results showed that all three materials presented same surface functional groups (Supplementary Figure S1). They could be considered as highly stable colloidal suspensions in the culture media, as their TSI values were found to be lower than five (Supplementary Figure S2). lGO and mlGO displayed similar lateral dimensions, compared to sGO (Figure 1 and Figure S3). However, mlGO presented thinner structures than the other materials. These results were also corroborated by X-ray experiments, since mlGO presented a non-defined crystalline character [18,25] (Supplementary Figure S4). In addition, they could be defined as low defect materials based on their Raman results (Supplementary Figure S5). Moreover, the results showed that lGO and mlGO produced more stress than sGO, indicating that the lateral dimension of the GO could be established as one of the main physicochemical properties to pay attention to for cell damage in this specie [18]. Therefore, the study analyzes—for the first time—the impact of these emerging GO nanomaterials, at the molecular level, on the gene expression in *C. riparius* after 96 h of exposure.

### 3.1. Stress Response

**Figure 2:**
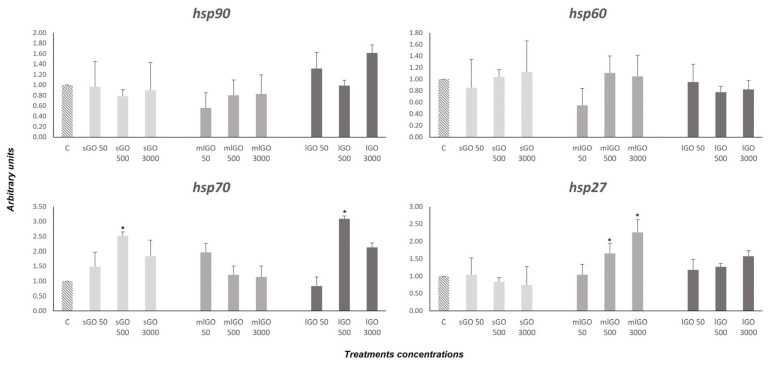
Expression of *hsp90*, *hsp70*, *hsp60* and *hsp27*, related to the stress response in *C. riparius* larvae after exposure to 50, 500 or 3000 μg/L sGO, mlGO and lGO for 96 h. Levels were normalized to control, which was set to 1. * Significant differences (*p* ≤ 0.05).

**Figure 3:**
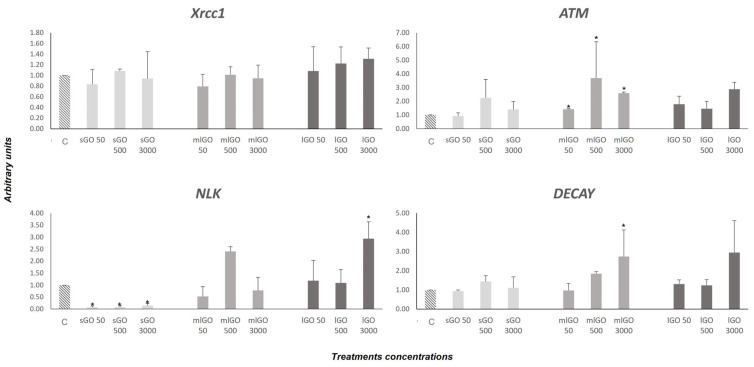
Changes in the mRNA expression of *XRCC1*, *ATM*, *NLK* and *DECAY* after 50, 500 or 3000 μg/L sGO, mlGO and lGO treatments for 96 h. The levels were normalized to control, which was set to 1. * Significant differences (*p* ≤ 0.05).

HSPs belong to a very old and conserved protein family responsible for maintaining cellular homeostasis in response to different external factors [38]. Mainly, HSPs function as chaperones in protein folding and unfolding. Although these proteins were discovered as stress response factors, HSPs are activated in various cellular processes, such as cell division and cell cycle, apoptosis, development and differentiation [39,40]. Different genes of this family have been characterized and analyzed in *C. riparius* in response to different anthropogenic pollutants (heavy metals, bisphenol A [BPA], tributyltin oxide [TBTO], pentachlorophenol [PCP], phthalates, multi-walled carbon nanotubes [MWCNTs], and microplastics [19,32,34,35,41,42]. In addition, HSPs are anti-apoptotic proteins [43,44] and include HSP90, HSP70 and HSP27. These proteins can block the cell death process at different points by interacting with proteins of the programmed cell death machinery [44,45]. In the study, the response of four stress response genes (*hsp90*, *hsp70*, *hsp60* and *hsp27*) was evaluated after exposure to three types of GO with different lateral dimensions and thickness. The *C. riparius* larvae were exposed to different GOs at various concentrations and times. The results showed a significant increase of *hsp90* mRNA expression by lGO (50 and 3000 µg/L); of *hsp70* by sGO (50, 500 and 3000 µg/L), mlGO (50 µg/L) and lGO (500 and 3000 µg/L); and of *hsp27* by mlGO (500 and 3000 µg/L) and lGO (3000 µg/L) (Figure 2). These results show that mlGO and lGO alter the cellular stress response more dramatically than sGO, suggesting that the lateral size of the material has an influence on the stress response of *C. riparius*. Previously, pure GO solutions and GO contaminated with manganese ions have been described to activate the *hsp70* gene in *Acheta domesticus* after 24 h of exposure [46,47], but there are not many other studies on the subject. However, as mentioned above, heat shock proteins are also anti-apoptotic proteins, and it is likely that up-regulation of the expression of these anti-apoptotic genes is the response to GOs-induced apoptosis. This would be supported by the increased mRNA levels observed for DECAY (Figure 3), a relevant effector caspase in programmed cell death processes during *Drosophila* development [48]. Other works have shown that MWCNT induces the expression of apoptosis-related genes in fish [49] and invertebrates such as *Caenorhabditis elegans* and *Chironomus riparius* [19,50].

On the other hand, the hsp60 chaperone is a protein involved in protein folding in mitochondria (Boshoff, 2015). However, there are no studies on how GO affects the *hsp60* mRNA expression. In larvae exposed to sGO, mlGO and lGO, no changes in the mRNA expression of this gene were observed under the conditions studied (Figure 2). These outcomes indicate that these materials do not affect mitochondrial protein folding at the times and concentrations tested.

### 3.2. DNA Damage Response

Previous studies have shown that excessive exposure to GO materials caused DNA damage due to ROS generation in human cells [51], fish cell lines [52,53], *D. magna* [22,54] and in *Danio rerio* [29,55]. However, the repair mechanisms for damaged DNA due to the GO exposure are still unknown, mainly in invertebrates. However, Lu et al. (2017) [12] showed that high concentrations of GO could cause DNA damage in human cells (HEK293T) and zebrafish embryos and proposed the base pair excision (BER) mechanism as a possible cellular response pathway.

In this work, we studied the transcriptional activity of *XRCC1*, *ATM*, *NLK* and *DECAY* genes, whose actions are related to the genotoxic effects derived from exposure to different compounds. These genes have been previously characterized and analyzed in *C. riparius* [36]. They are involved in different DNA repair mechanisms and the resulting expression pattern demonstrates a variety of damage. The *XRCC1* gene encodes for a BER enzyme that acts similarly to cellular endonucleases. The XRCC1 enzyme is implicated in single-strand break-repair processes and is also involved in the subsequent restoration of the cleaved sequence [56,57]. With the conditions established in our study, no modifications in the *XRCC1* gene mRNA expression were observed after larvae treatment to the GOs studied (Figure 3). Therefore, we can conclude that GO nanomaterials do not activate this specific DNA repair mechanism through the *XRCC1* gene, at least under the conditions selected. On the other hand, ATM is a protein kinase implicated in the response to DNA double-strand breaks (DSBs) and has also been shown to have several very relevant functions in the cell, since its mutation or inactivity causes serious pathologies, such as oxidative stress or mitochondrial dysfunction [58,59]. In this study, *ATM* gene expression is upregulated after exposure to all the mlGO concentrations employed, as well as to lGO at 3000 µg/L and sGO at 500 µg/L (Figure 3). This would mean the graphene nanomaterials can cause damage to cellular DNA and alter the activity of this gene, hence increasing its expression levels. In this regard, some studies have shown that *ATM* acts as a mediator in the regulation of the global cellular response to oxidative stress [60]. Our results for ATM upregulation are consistent with the increased mRNA expression of the *hsp* 90, 70 and 27 stress proteins. They would support the idea that GO nanomaterials can produce oxidative stress in addition to inducing DNA damage in *C. riparius*. There are no previous studies that have analyzed the activity of the *ATM* gene upon exposure to graphene nanomaterials; therefore, our results are the first piece of evidence that this type of carbon-derived compound alters the expression of this important gene involved in DNA repair.

DECAY is a class II effector caspase that shares homology with mammalian caspases 3 and 7, whose expression in vitro in cells induces apoptosis. In *Drosophila*, it appears to be involved in programmed cell death processes during development [48]. The results obtained here showed that the *DECAY* expression is activated after 96 h of exposure to mlGO (500 µg/L and 3000 µg/L) and lGO (3000 µg/L) (Figure 3). However, exposure to sGO does not produce any change in the transcriptional activity of this gene. Therefore, we can conclude that mlGO and lGO are able to induce apoptosis in *C. riparius* under the conditions tested in contrast to sGO. There is no previous evidence of *DECAY* gene disruption with exposure to graphene materials.

NLK belongs to the family of NEMO-like kinases (NLKs), proteins responsible for the regulation of different signal transduction pathways (Ishitani and Ishitani, 2013). All the concentrations of sGO produced an overall inhibition of the *NLK* gene compared to mlGO and lGO, resulting in transcriptional activation of this gene. Recent studies have shown that NLK is also involved in DNA repair mechanisms since it is required for the activation of the p53 protein in response to genotoxic damage [44]. Non-activation of the *NLK* gene would result in inactivation of p53, preventing activation of cell apoptosis at that point. These results would coincide with the non-alteration of the DECAY apoptosis-promoting caspase expression levels obtained after exposure to sGO. Taken together, this may indicate that the sGO nanomaterial would be less damaging to the cell compared to mlGO and lGO, since it does not cause apoptosis. Similar results for *NLK* gene and *DECAY* inactivation have been obtained previously in *C. riparius* after treatment to the fungicide Vinclozolin [36]. In addition, the results obtained with *mlGO* and *lGO* could suggest that the activation of the repair mechanisms produced after their exposure in the *C. riparius* larvae would not be sufficient to stop cell apoptosis. The activation of apoptosis could be a consequence of the activation of repair mechanisms due to the DNA DSBs induced by these NMs. These results are the first example of evidence for the ability of GOs to activate apoptosis. Other works have shown that MWCNT induces the expression of apoptosis-related genes in fish [49] and invertebrates [19,50].

In summary, our results have shown that all the studied GOs modified the transcriptional activity of the *ATM* and *NLK* genes related to DNA damage repair, while the mlGO- and lGO-triggered apoptosis was mediated by the activation of the DECAY caspase. However, the GOs did not induce the expression of the *XRCC1* gene implicated in the DNA single-strand damage repair. Both DNA repair genes, *ATM* and *DECAY* were affected according to the concentration of the compounds used. These results are further evidence that GO exposure produces genotoxic damage, leading to activation of DNA repair genes in *C. riparius*.

Further research analysing the other genes involved in this mechanism will be necessary to obtain an overall picture of the DNA damage caused by these graphene oxide-based materials.

### 3.3. Antioxidant, Endocrine and Developmental Responses

Oxidative stress (*SOD Mn*) and developmental (*FKBP39*) and endocrine (*DRONC*) genes were assessed. *SOD Mn* synthesizes a protein that protects the cell from oxidative stress [61]. In this study, *SOD Mn* was significantly upregulated in the *C. riparius* larvae exposed to 500 and 3000 µg/L mlGO. Although the larvae exposed to lGO showed a tendency to increase the expression, it was not significant. The results indicate that mlGO causes oxidative stress and SOD Mn is activated to mitigate the effects. These results agree with those previously obtained through analysing the enzymatic activity of SOD [18] in response to GOs in *C. riparius*. Signs of *SOD Mn* in response to GOs have been detected in aquatic organisms [18].

In general, responses to oxidative stress may vary because of differences in the lateral size and thickness of materials.

**Figure 4:**
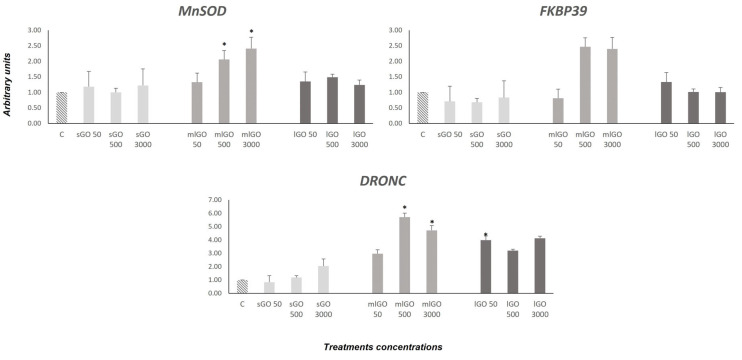
Changes in the mRNA expression of *MnSOD* (oxidative damage response), *DRONC* (endocrine system) and *FKBP39* (development). Levels were normalized to control, which was set to 1. * Significant differences (*p* ≤ 0.05).

FKBP39 is involved in growth and developmental processes. This protein acts as an expression modulator in the 20-hydroxyecdysone and juvenile hormones in *D. melanogaster* [62,63]. *FKBP39* was upregulated to 500 and 3000 µg/L (mLGO) (Figure 4). Considering the results obtained, GOs could affect the growth and development of *C. riparius* larvae. Moreover, FKBP39 was described as a physiological inhibitor of autophagy in *D. melanogaster* [64]. This inhibition is mediated through the insulin receptor (Foxo) pathway. It is an endocrine pathway gene involved in the response to insulin-like peptides. The overexpression of FKBP39 occurs early in the *Drosophila* development [65]. In this regard, mlGO appears to impact the development and endocrine system of *C. riparius*.

Finally, an increase in the *DRONC* expression was demonstrated in the larvae exposed to sGO (3000 µg/L), mlGO (50, 500 and 3000 µg/L) and lGO (50, 500 and 3000 µg/L) as endocrine disruptors (EDCs) in chironomids. *DRONC* is a caspase described in *Drosophila* [66] that is involved in insect metamorphosis, acting as an effector gene, downstream of the ecdysone receptor (EcR) in the 20-E signalling pathway [49]. The increased expression of *DRONC* could be due to the prior activation of *EcR*. Results obtained seem to indicate that modifications at the molecular level of the endocrine system could be related to the developmental alterations already observed in *C. riparius*. However, to confirm this hypothesis, it would be necessary to evaluate other genes related to metamorphosis in invertebrates.

## 4. Conclusions

The results show that the lateral dimension and thickness of GO flakes are a fundamental physicochemical parameter in the toxicity of these nanomaterials in *C. riparius*. mlGO and lGO, materials of greater dimension and thickness than sGO, produce effects on the expression of genes of different metabolic pathways (cellular stress, DNA repair genes, oxidative stress, developmental and endocrine stress). The results suggest that mlGO and lGO produce oxidative stress (ROS) that activates antioxidant mechanisms (SOD) and, in turn, could cause DNA damage, as shown by the transcriptional modification of ATM and NLK genes. Furthermore, the induction of the anti-apoptotic chaperone genes hsp90, hsp70 and hsp27, together with the overexpression of the apoptosis-inducing gene DECAY, suggest an activation of apoptosis generated after exposure to these two nanomaterials.

In addition, genes related to the endocrine system and to the development of *C. riparius* are modified by these nanomaterials. Future studies should delve deeper into the pathway of toxicity on the endocrine and developmental system.

In conclusion, this work demonstrates for the first time that these graphene nanomaterials affect the transcription of genes involved in different metabolic pathways in the aquatic invertebrate *C. riparius*. From the perspective of the synthesis and utilization of carbon nanomaterials, our data have shown that the lateral dimension of this type of carbon-based nanomaterials is one of the most important properties to be paid attention to in future research.

## Figures and Tables

**Table 1:** Oligonucleotides sequences.

Gene	Primers	Process	
***rpL13F***	ACCAGCTAGAAAGCACCGTC		[32]
***rpL13R***	ATGGGCATCTGACGATTGGG		
***GAPDHF***	GGTATTTCATTGAATGATCACTTTG		[33]
***GAPDHR***	TAATCCTTGGATTGCATGTACTTG		
***hsp90F***	AGGCTGAAGCTGACAAGAATG	Stress response	[34]
***hsp90R***	TCATGCGATAAATGCGAGCAG		
***hsp70F***	ACTTGAACCAGTTGAGCGT	Stress response	[27]
***hsp70R***	TTGCCACAGAAGAAATCTTG		
***hsp60F***	TGCTGTCCTTAAAGTCGGTGG	Stress response	[35]
***hsp60R***	TCCACCACC-CAACGATTC		
***hsp27F***	TCAACACACAGGACCG	Stress response	[30]
***hsp27R***	ATCCTTTATTGGTGATTAATTATG		
***XRCC1 F***	GACGATTTGCATTGGATAGT	DNA repair. SSB/DSB	[36]
***XRCC1 R***	ATCAACATATCGCCATCAG		
***ATMF***	ACATTTGGCGTAGATCAGGCA	DNA repair. SSB	[36]
***ATM***	ACGAGATGCATCAAATCATGC		
***NLKF***	CATCTCACCAGATCGTCTCT	DNA repair. SSB	[36]
***NLKR***	GAATTTATTTGATTATGCGGC		
***DECAY F***	AAAGTGTTCCGATTATGGC	DNA repair. Apoptosis	[36]
***DECAY R***	TTCACACCAGTTAAAATCCAC		
***SODMnF***	AAGTCGCTGCTGTTGGAGTT	Oxidative stress	[34]
***SODMnR***	TGGAACTAAGCCGGTTGTGG		
***FKBP39F***	AGGCTGGGATATCGGACTCAT	Development	[34]
***FKBP39R***	GTAAGCAAATGCAGGCGGG		
***DRONCF***	GAAATGTCACAGATTTCAGTGCC	Death regulator Nedd2-like caspase	[37]
***DRONCR***	GTGAATATCGTAAGCATGTTCTGC		

## Data Availability

All the data obtained in this work are available at: https://unedo365-my.sharepoint.com/personal/mfolgar_ccia_uned_es/_layouts/15/onedrive.aspx?id=%2Fpersonal%2Fmfolgar%5Fccia%5Funed%5Fes%2FDocuments%2FGraphene%20Oxides%20%20%20%28GOs%29%20with%20Different%20Lateral%20Di%2Dmensions%20and%20Thicknesses%20Affect%20the%20Molecular%20Response%20in%20%20Chironomus%20riparius&ga=1 (accessed on 3 March 2023).

## Supplementary Materials

The following supporting information can be downloaded at: https://www.mdpi.com/article/10.3390/nano13060967/s1, Figure S1: Surface chemistry of the different GO materials determined by FTIR-ATR. Figure S2: Colloidal stability of the different GO materials measured with Turbiscan. Figure S3: Lateral dimension histogram for sGO material. Figure S4: Raman spectra and the ID/IG values of the different GO materials. Figure S5: XRD spectra of the different GO materials. Figure S6: Pathways and corresponding genes affected by different GO materials. Figure S7: Proposed adverse outcome pathway for NP toxicity related to endocrine and neurotoxic effects.
