# Supplementary material for: Graphene Oxides (GOs) with Different Lateral Dimensions and Thicknesses Affect the Molecular Response in Chironomus riparius
Source: Nanomaterials (Basel). 2023 Mar 7;13(6):967. doi: 10.3390/nano13060967 (PMC10057717; doi:10.3390/nano13060967)
Supplement: Supplementary file 1 [file nanomaterials-13-00967-s001.zip › nanomaterials-2189502-supplementary.pdf]

nanomaterials

Supplementary Materials

Graphene Oxides (GOs) with Different Lateral Dimensions and Thicknesses Affect the Molecular Response in *Chironomus riparius*

Raquel Martin-Folgar [1,*], Adrián Esteban-Arranz [2], Viviana Negri [3] and Mónica Morales [1]

[1] Grupo de Biología y Toxicología Ambiental, Departamento de Física Matemática y de Fluidos, Facultad de Ciencias, UNED, Urbanización Monte Rozas, Avda. Esparta s/n, Crta. de Las Rozas al Escorial Km 5, 28232 Madrid, Spain; mmorales@ccia.uned.es
[2] Instituto de Ciencia y Tecnología de Polímeros (ICTP-CSIC), C/ Juan de la Cierva 3, 28006 Madrid, Spain; adrian.esteban@ictp.csic.es
[3] Departamento de Ciencias de la Salud de la Universidad Europea de Madrid (UEM), C/ Tajo, Villaviciosa de Odón, 28670 Madrid, Spain; viviana.negri@universidadeuropea.es
* Correspondence: mfolgar@ccia.uned.es; Tel.: +34-913987124

List of Figures

Figure S1. Surface chemistry of the different GO materials determined by FTIR-ATR.
Figure S2. Colloidal stability of the different GO materials measured with Turbiscan.
Figure S3. Lateral dimension histogram for sGO material.
Figure S4. Raman spectra and the I_D/I_G values of the different GO materials.
Figure S5. XRD spectra of the different GO materials
Figure S6. Pathways and corresponding genes affected by different GO materials
Figure S7. Proposed adverse outcome pathway for NP toxicity related to endocrine and neurotoxic effects.

Characterization results of Graphene Oxide materials

Figure S1 shows the surface chemistry of the different materials. As a first conclusion, it can be seen that all of them present similar surface functional groups. The band detected between 3680 cm^{-1} and 2910 cm^{-1} and the band located at 1053 cm^{-1} evidence the presence of hydroxyl groups and epoxy groups, respectively. Moreover, the band at 1723 cm^{-1} is characteristic of the carbonyl group from carboxyl groups. The sp^2 hybridization typical of graphenic materials is shown with the bands at 1400 cm^{-1} and 1625 cm^{-1}.

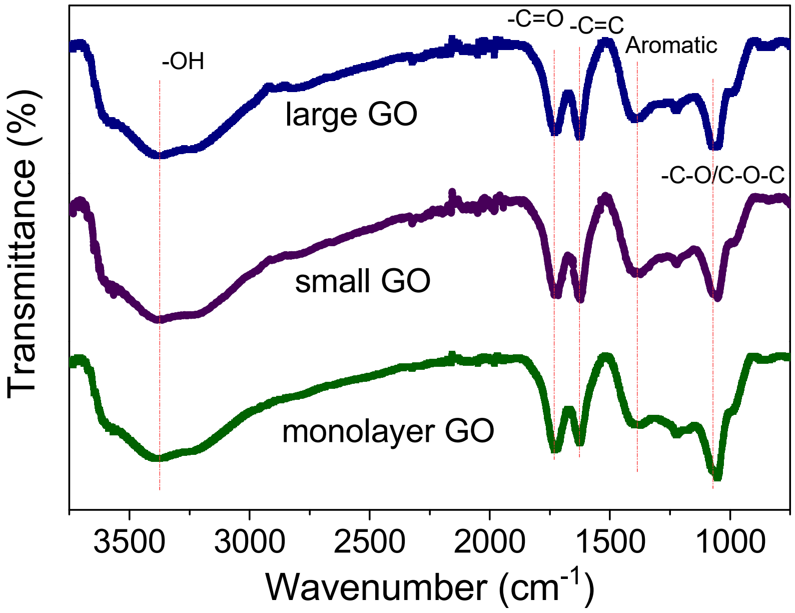

Figure S1. Surface chemistry of the different GO materials determined by FTIR-ATR.

Turbiscan experiments were conducted to elucidate the colloidal stability of these materials in the culture media of the larvae. Results are presented in Figure S2. They are consistent with the outcomes of Martin-Folgar et al., 2022 in that the TSI values obtained by these GO materials are lower than five (lGO= 4.2±0.3, sGO= 2.7±0.2 and mlGO= 2.35±0.2). Based on Dai et al.'s classification, these values are characteristic of highly stable GO materials, the monolayer graphene oxide being the most stable material in this culture media.

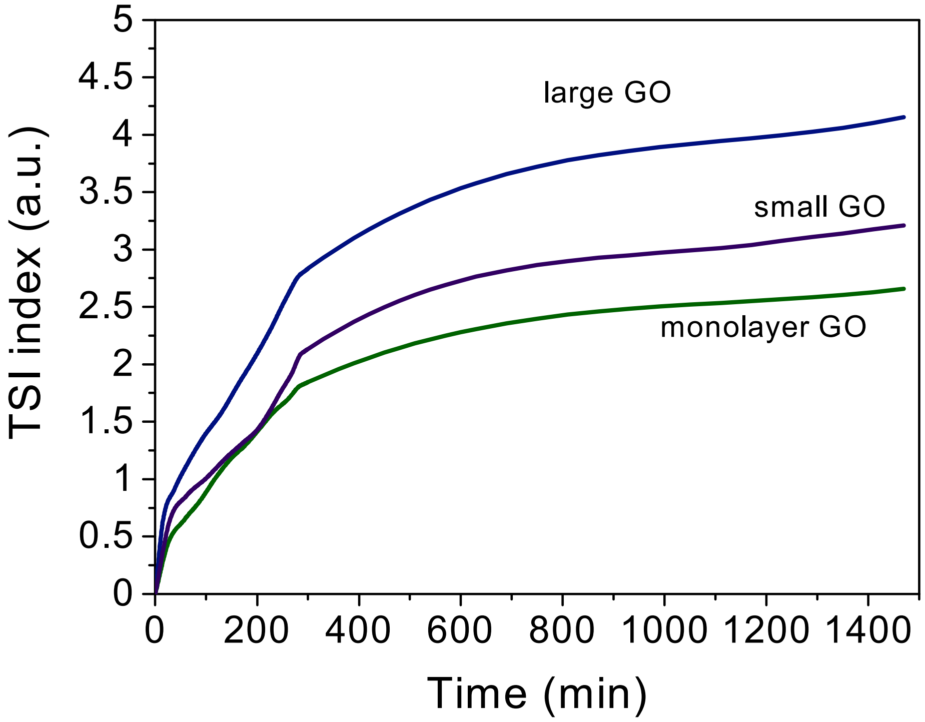

Figure S2. Colloidal stability of the different GO materials measured with Turbiscan.

A study on the lateral dimension of sGO was carried out. Figure S3 shows that sGO mainly display lateral dimensions between 0.8 to 1.5 μm. These results are in accordance with previous outcomes of the studies by Martin-Folgar et al. and Esteban-Arranz et al.

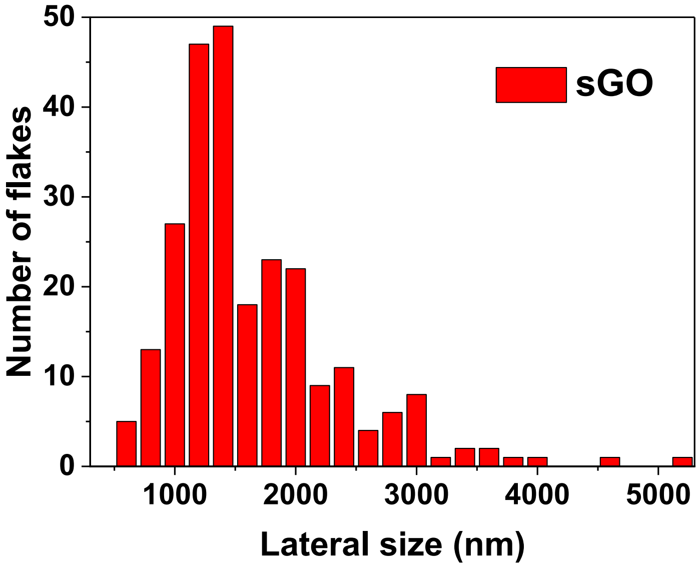

Figure S3. Lateral dimension histogram for sGO material.

Raman spectroscopy experiments were carried out to define the structural properties of these GO materials. Their spectra are depicted in Figure S4. In addition, their I_D/I_G ratio values, presenting their degree of defects, are also included. These GOs display I_D/I_G values between 1.30-1.37, characteristic of this kind of material. It can be seen that mlGO exhibits a lesser degree of defects than lGO and sGO. Based on D. López-Díaz's studies, they can be considered as low-defect graphene-based materials, since their I_D/I_G ratio values are lower than 3.5.

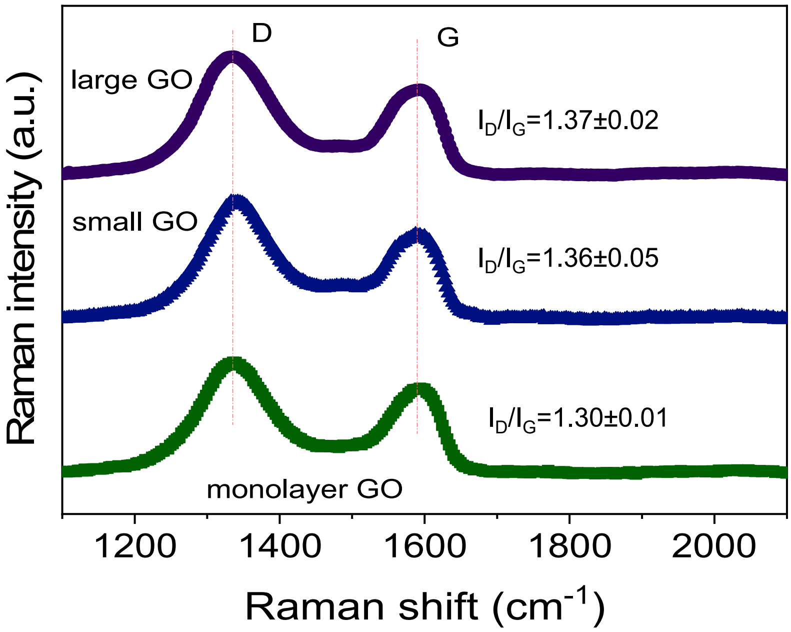

Figure S4. Raman spectra and the ID/IG values of the different GO materials.

Figure S5 shows the XRD results obtained for these GO materials. After the oxidation process, oxygen functional groups are incorporated. This partial change of hybridization from sp^2 (graphite) to sp^2-sp^3 (graphene oxide) can be evidenced by XRD. The peak, referred to the (001) plane, that is found in lGO and sGO diffractograms indicates this structural modification. Both GO materials present some graphitization degree, possible due to their greater number of graphene oxide layers. However, mlGO displays a flatter diffractogram in contrast to sGO and lGO, which is representative of a more exfoliated graphene oxide material.

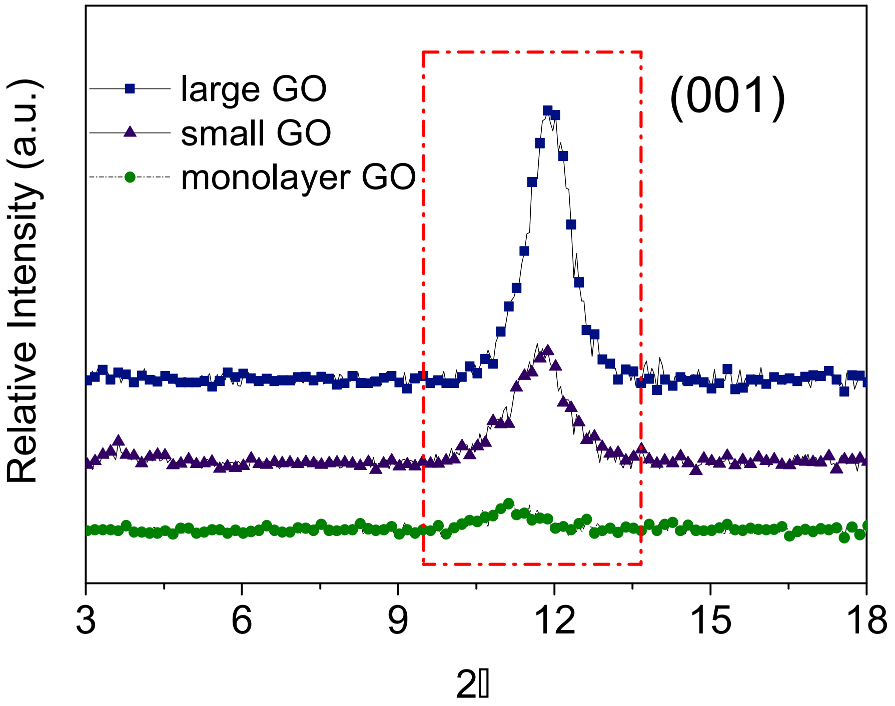

Figure S5. XRD spectra of the different GO materials.

Figure S6 shows an outline of the pathways and corresponding genes that are affected by each material. Black arrows indicate overexpression and red arrows indicate inhibition of gene expression.

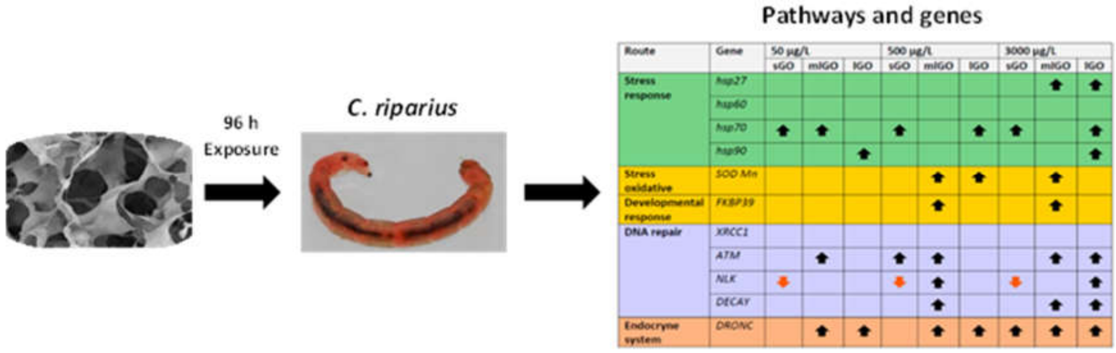

Figure S6. Pathways and corresponding genes affected by different GO materials.

Figure S7. Proposed adverse outcome pathway for NP toxicity related to endocrine and neurotoxic effects.

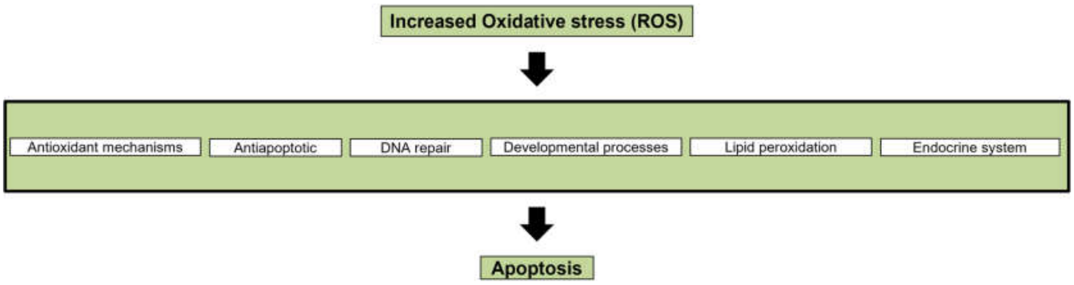

Figure S7. Proposed adverse outcome pathway for NP toxicity related to endocrine and neurotoxic effects.
